# Between Sympathy, Fascination, and Powerlessness. The Experiences of Health Professionals During the Medical Monitoring of a Hunger Strike Among Undocumented Migrants

**DOI:** 10.3389/fpubh.2022.756964

**Published:** 2022-05-25

**Authors:** Rita Vanobberghen, Dirk Lafaut, Fred Louckx, Dirk Devroey, Jan Vandevoorde

**Affiliations:** ^1^Department of Family Medicine and Chronic Care, Vrije Universiteit Brussel, Brussels, Belgium; ^2^Department of Philosophy, Vrije Universiteit Brussel, Brussels, Belgium; ^3^Department of Sociology, Vrije Universiteit Brussel, Brussels, Belgium

**Keywords:** hunger strike, undocumented immigrants, ethical dilemmas, secondary traumatic stress, health profession

## Abstract

In 2014, a group of undocumented migrants started a hunger strike in Brussels. The medical monitoring was mainly done by young, committed health professionals with no prior experience of medical monitoring of people on hunger strike. Following the hunger strike, two focus groups were organized to assess the experiences of the health professionals during the medical monitoring of the hunger strike. Their main motivation for assisting was wanting to help the people on hunger strike but they were also curious about the living conditions among undocumented migrants and the reasons behind starting the strike. They were puzzled by the paradox of hunger strikers putting their life at risk in order to get a better life and obtain a residence permit. They felt conflicted about their own role as a caregiver: they did not know how to deal with patients who did not comply with medical advice, they struggled to build a relationship of mutual trust and feared that they would end up being instrumentalized by the hunger strikers or their environment. Afterwards, some of the health professionals were deeply touched by the experience and there were reports of symptoms of secondary traumatic stress such as re-experiencing and avoidance. During the focus group's discussions, the respondents made suggestions on how to improve the medical monitoring in the event of any future hunger strikes.

## Introduction

The struggle of undocumented migrants in Belgium has included many hunger strikes. This is done as Belgian law states that an undocumented migrant can request a temporary residence permit, initially for 3 months, if they are suffering from “*an illness which poses a real threat to their life or physical integrity or which implies a real risk of inhuman or degrading treatment when there is no adequate treatment in their country of origin or residenc*e” (1). The undocumented migrants' refusal to eat solid foods and instead only consume sweetened beverages meant that their health deteriorated until the point that negotiations with the Immigration Office led to them being granted a 3 month residence permit for the purposes of regaining their strength. The only alternative way to obtain a permanent residence permit was by presenting an employment contract, which is hard to come by as to be accepted, the contract must be for well-paid, fulltime employment in a profession where there are otherwise skill shortages in Belgium. There are also loopholes for the employer. For example, if the employee becomes ill, the employers have to cover all expenses such as hospital bills, sick leave and even repatriation in the event of death. A thorough screening of the company's financial viability is also carried out. In other words, obtaining such a contract is extremely difficult (2).

Instead of giving up, some undocumented migrants resort to hunger strikes to escape a life of misery and incertitude (3). Hunger strikers are defined as persons “*who undergo a substantial period of voluntary total fasting for a specific purpose”* (4). Studies of hunger strikes in prisons also mention food refusers: the noisy, angry prisoners who make a big fuss about small issues and have no intention to jeopardize their health are called reactive food refusers, while the determined food refuser is quite the opposite: he or she will stay silent about the fasting, that is rather a cry for help to get attention to a hopeless situation. Studies have highlighted that hunger strikers are not suicidal but that they are willing to put their wellbeing at risk to achieve their goals (4–6). Between 2008 and 2015, 1,158 hunger strikers from more than 18 countries participated in Brussels, in 15 different hunger strikes. In 2009, due to consecutive hunger strikes, the Belgian government implemented a 3 months period where undocumented migrants could apply for asylum under special terms. More than 40,000 people took advantage of this offer (7).

The medical monitoring of a hunger strike in custodial settings (prisons, detention centers and hospitals) has been extensively documented in literature. The most important guidelines are described in the 2006 Declaration of Malta (8, 9). It states that health professionals should address hunger strikers with respect, recognize their autonomy, give proof of clinical impartiality, and carefully observe confidentiality to gain and retain the trust of the participants. The physician's primary obligation is always to the individual patient, not to the institution employing him/her (9). Where they seek medical supervision, strikers should be free to choose whether and from whom they want medical care, including an independent, external physician (10, 11). Force feeding is classified by the WMA as an inhuman and degrading treatment that is never ethically, legally, or medically justified (12).

There are different ways in which a health professional can be involved with hunger strikers. Health professionals employed by custodial institutions can rely on an existing medical structure with a multidisciplinary, paid staff who will be able to monitor the participants to the hunger strike 24 hours a day. They can make use of established clinical guidelines, have enough medical equipment and access to laboratory analysis and radiological imaging. Usually the number of hunger strikers is limited and their medical history is known to the staff (10, 13–24). They will be confronted with the problem of divided loyalty. Should they obey the guidelines of the authorities responsible for the health of the inmates and prefer to force-feed them to keep them alive rather than to respect the strikers' decision to refuse food until death? Will they act as a tool for monitoring for government institutions by retrieving data and information and passing this on to controlling institutions? Or should they treat and give advice to the hunger strikers on how to stay as healthy as possible during the hunger strike? Salas et al. (25) argue that hunger strikers must be informed beforehand of the role of the visiting health professionals (25).

Even though most studies of hunger strikes are done in prisons, jails or detention centers, 30% of hunger strikes are held in non-custodial settings (5). This includes farmers fighting to preserve their land, religious minorities subject to harassment or discrimination, miners who think they will lose their jobs and livelihoods, or people who fear being denied their basic rights because of their political or social associations. Undocumented migrants and asylum seekers also have a long history of fighting inhumane living conditions they have to endure. Undocumented migrants went on hunger strike to protest against the demolition of the “jungle” of Calais in France and the horrible living conditions in the reception centers on the Greek and Australian islands. Afghan refugees protested in front of the UN High Commissioner for Refugees in Ankara (Turkey) because they were denied their basic rights (26). Given that these forms of resistance very often arise spontaneously and are neither orchestrated nor prepared, few scientific studies have been carried out. It was our hypothesis that the experiences of health professionals in such hunger strikes differ from the experiences in custodial settings as described above. In non-custodial settings, the hunger strikers themselves can choose from whom they seek assistance and can find independent health professionals to do the monitoring or follow-up. This means giving them advice about what to drink, which medicines or vitamins to take, monitoring their medical parameters and intervening when necessary. This assumes a more mutual respectful relationship between doctor and patient (27). These health professionals are less likely to be caught up in monitoring such as verifying the authenticity of the hunger strike, but rather support the patient and intervene before irreversible bodily damage (such as the Wernicke-Korsakoff Syndrome from a lack of vitamin B 1) occurs.

To this end, we studied the experiences of health professionals who volunteered to provide medical assistance during a hunger strike in a non-custodial setting in Belgium. This article is part of a wider doctoral research project on this subject. Previously published articles focus on the socio-economic impact of the hunger strike on the participants 5 years later and on the special role of health professionals during a hunger strike in a non-custodial setting (28, 29). The aim of the present study is to describe the motivations, the experiences and the concerns of health professionals during the two-month medical monitoring of a hunger strike of undocumented migrants in Brussels and to describe their suggestions for improvement of medical monitoring during a hunger strike.

## Study Context and Methodology

In 2014, a group of about 200 undocumented migrants, mostly families with children, were squatting in a vacant nursing home in a suburb of Brussels. Some of them demonstrated weekly in front of the Immigration Office to demand a more humane treatment and a residence permit. Following a lack of response, 45 of them started a hunger strike on 17 November 2014. The participants to the hunger strike came from Morocco, Guinee, Guinee Bissau, Burkina Faso, Ivory Coast, Cameroon, Senegal, Mali, Mauretania, Turkey and India. Five of them were women, who occupied a separate room together with their children. The 40 men, mostly between 20 and 30 years of age all slept together in the main room on the ground floor, without any privacy. From the start there was friction between the group on hunger strike and the non-striking migrants.

The partial hunger strike, with the intake of sweetened beverages, lasted 64 days. Shortly after starting the strike, activists requested medical assistance by contacting the main researcher (RV), a medical doctor in Family Medicine, who was known to have assisted individuals during previous hunger strikes. The main researcher (RV) contacted several other health professionals through informal networks and with a call on social media, together forming a group to perform medical monitoring. The group consisted of two nurses, 10 medical students and 17 medical doctors, of whom eight at the time were in training in Family Medicine or Internal Medicine. Most of them worked in multidisciplinary community health centers in the suburbs of Brussels and had never monitored a hunger strike before; only one specialist and the main researcher (RV) had previous experience with medical assistance during a hunger strike. A schedule was made to organize home-visits to the undocumented migrants on hunger strike twice a week. As most undocumented migrants had been in Belgium for several years, most conversations were held in French and sometimes in English. Students and junior doctors were always supervised by a senior health professional. The health professionals did not receive any remuneration for their participation.

The medical monitoring entailed the measuring of vital signs, as well as the treatment of physical and psychological complaints. Any suggestions for treatment were noted in the medical records of each patient. All undocumented migrants on hunger strike were encouraged to drink more than 1.5 liters of fluids daily, take stomach protection (Proton Pump Inhibitors) and a vitamin B1 pill. In order to prevent dehydration or electrolyte disturbances, the intake of Oral Rehydration Serum (O.R.S.) was recommended. This can be homemade but has a very salty, unpleasant taste. Commercial salt tablets being too expensive, the participants to the hunger strike made their own salt capsules by putting 1.5 g of kitchen salt in an empty hard gelatin capsule with the help of a “capsule filling machine” (16, 20, 23). In addition to the individual notes in the medical records, each team also prepared a handover sheet for the next team of health professionals. They noted the bed numbers of patients who needed extra attention. Twice a week, medical updates were sent out to the health professionals. This consisted of anonymized lists of weights, BMI and, if relevant, bed numbers of patients transferred to hospitals.

Initially, most hunger strikers complained of gastro-intestinal problems as well as dizziness, headaches and extreme fatigue and in addition, many reported psychological problems. As the hunger strike progressed and fat reserves were used up, the complaints changed to body aches, kidney problems and neurological symptoms. Access to public healthcare coverage was only possible if the person had gone through a complex procedure of seeking “urgent medical aid”, however most of the hunger striking migrants did not seek such aid. This meant that buying drugs or carrying out medical testing such as urine or blood exams on the site was almost impossible (28). Patients who were critical were transferred to the emergency ward of nearby hospitals where they could be given more thorough medical assessments and as well as IV fluids where indicated.

In January 2015, following negotiations with the Immigration Office, the hunger strike ended. The undocumented migrants who had participated in the hunger strike and become very weak, only obtained a feeble promise that, if they re-applied for asylum, their records would be given priority. Two weeks after the strike ended, the building was evacuated by the police, the occupiers were dispersed across the whole city and there was no follow-up of the outcome of the asylum procedure. It is unclear to the authors what happened to any of the migrants after this, but it is likely that they went back to an illegal stay, crossed borders to seek asylum in another European country or were deported.

A few days after the hunger strike ended, all health professionals involved in the medical assistance during the hunger strike were invited to participate in focus group discussions. Our main reason for these discussions was that it allowed us to collect data from many health professionals shortly after the hunger strike, when the memories about it were still fresh and detailed. The use of focus group of peers who had shared a similar experience also created a safe environment for the health professionals to express their experiences. This approach also allowed us to learn more about group attitudes of and interactions between different health professionals. The research was approved by the Medical Ethics Committee UZ Brussel[s]—VUB (B.U.N. 143,201,940,934). All respondents agreed to participate, and filled in and signed a consent form. No respondents received financial incentives.

Eighteen health professionals consented to participate. Two focus group meetings were organized. The first took place a few days after the hunger strike ended, another one was held 1 month later. The first meeting was attended by 10 people (three medical students, one nurse and six doctors, of whom four in training), the second had eight participants (two students, one nurse and five doctors, of whom three were in training). For all but one of the responding health professionals it was their first time assisting patients during a hunger strike. All respondents had experience of working with deprived groups in multidisciplinary clinics or hospitals in Brussels (Table 1). Both focus groups were facilitated by two researchers, both staff members of departments of Family Medicine. Both researchers had experience in providing medical assistance to people on hunger strike. Both focus group discussions lasted about 2 h. The discussions were recorded and the recordings were transcribed and analyzed in NVIVO by the main author (RV).

**Table 1:** The experiences of health professionals while monitoring a hunger strike among undocumented migrant workers.

**Characteristics of the participants**	
**Gender**	
Man	8
Woman	10
**Age**	
19–29	12
30–49	4
>50	2
**Profession**	
Medical student	5
Nurse	2
Junior doctor	7
Medical doctor	4

Four main topics were discussed. Firstly, what were the main motivations for engaging in the medical monitoring around the hunger strike? Secondly, how did the health professionals feel during and after the home visits and the consultations? Particular attention was paid to anything that stood out, what had bothered them, and what dilemmas they had experienced during the actual caregiving. Questions also explored the impact of the medical assistance of the hunger strike on their own lives. Thirdly, how did the health professionals cope with the challenges that they had experienced? Lastly, did the health professionals have any suggestions for improving medical supervision of any future hunger strikes?

## Results

### Soothing the Health Professional's Curiosity, Fascination, and Political Sympathy

When asked about their motivation to engage in the medical assistance of the hunger strike, two nurses responded:

*It's an experience that you can give yourself to measure the level to which people can put themselves in danger… and you can go to there to feel it yourself, you can experience it yourself (Nurse)*.

*We are really getting out of the normal routine, and we are not even forced to do it (Nurse)*.

The health professionals, and in particular the young ones (i.e., the students and the doctors in training), explained that their engagement was a way to expose themselves to a completely novel experience, a way of getting out of the routine, a way to feel the despair of individuals who start a hunger strike. This expectation was also fulfilled: they expressed later in the discussion that the atmosphere had been completely different from what they were used to, even if they had been in contact with undocumented migrants during their medical practice before. Furthermore, the first quote also illustrates that the health professionals were simultaneously intrigued and puzzled, but also attracted by the paradox embodied by the hunger strikers: the paradox of wanting to live but simultaneously putting themselves in danger and risking death for a cause.

In the focus group discussions, this led to diverging views on whether the participants to the hunger strike wanted to live or die. Many health professionals expressed that the undocumented migrants on hunger strike preferred to live, even if they said they wanted to continue the hunger strike till the bitter end. They argued that if they wanted to die, they would not have accepted the presence of medical personnel. Being confronted with the definition of hunger strikers and food refusers some health professionals suggested that the participants to the strike were rather “determined food refusers”: persons who have lost all hopes and see no other solution than slowly disappearing. Meaning that a different medical approach of treating an imminent depression was more appropriate. Some of the respondents even recognized “reactive food refusers”: very vocal, angry participants who made a lot of fuss, but who were very worried about their wellbeing and had no intention to endanger their health (4).

Yet, an older medical doctor, who had monitored hunger strikes in the past, expressed doubt:

*I do not know if some are not ready to die, when you see those who cross the Mediterranean Sea, on inflatable rafts, knowing that so many before them drowned on the way and they do it anyway (Medical doctor)*.

The doctor draws parallels between a hunger strike and the migration trajectory. There is a risk, but it is a well-considered risk the undocumented migrants are willing to take to reach a final destination, a final goal. A junior doctor agreed that they might be willing to die, but described the ultimate goal as more political, doing something horrific to denounce their living conditions in Belgium:

*They continue the struggle even if they are weak: by dying they can prove the horror of their situation (Junior doctor)*.

In addition to being fascinated by the paradox of a hunger strike, health professionals were also curious about more practical issues. Respondents described how they, during their work, had been fascinated by whether the hunger strike eventually would be successful, that is whether the demands of the strikers would be met. They also exchanged views on how they thought the hunger strike started: had one person suggested starting a hunger strike and had the rest followed? How could they reach a joint decision? Why did most of the participants have the same profile (male, between 20 and 30, with African roots)? One health professional wondered if the request for medical monitoring had come from the participants to the hunger strike or from the outside. The health professionals also wondered how they eventually had decided to stop the strike.

On further probing on the motivation to engage in the medical supervision of the hunger strike, the health professionals stated that with their presence they expressed sympathy with the political demands of the strike.

*Our presence proves that we support them (Junior doctor)*.

*What is stupid to start with, is that people have to go on a hunger strike to be heard (Medical doctor)*.

They stated that if there had been other, legal ways of obtaining a residence permit, no-one would have started a hunger strike. They expressed awareness and condemnation of the poor socio-economic situation of undocumented migrants. The health professionals also explicitly linked their own motivations to provide medical care to the motivations of the individuals who were on hunger strike. In doing so, the health professionals made a number of assumptions about the underlying reasons of the undocumented migrants for starting a hunger strike and found that they were justified. They believed that the migrants had done it to obtain residence papers, to be heard and to fight against unfair migration policies and that they also had realized that other undocumented migrants in the past had obtained papers by going on hunger strike and that the same strategy might work for them too,. Others described the societal exclusion of undocumented migrants and the shame they would experience upon return to their countries of origin, if they would have to confess that their attempt to get a better life in Europe had failed, especially when acquaintances had contributed financially to their journey.

### Being a Good Health Professional

The health professionals pointed to several challenges and concerns regarding their own role during the hunger strike. First of all, several health professionals had felt overwhelmed and expressed a sense of powerlessness.

*As health professionals we cannot do much, it's very difficult to let people deteriorate (Junior doctor)*.

*What can we bring them with our limited means that they will accept, what can we do to help them? (Medical student)*.

The quotes illustrate the difficult, conflicted feelings of the health professionals during the hunger strike. They felt like they were “letting deteriorate”, or “not doing much”, or “not bringing anything”. The doctor also uses the word “people”, in plural. This illustrates another concern that was raised during the focus group discussions. During the hunger strike, it was difficult to give patients an individualized treatment, they were seen as a group. Respondents said that consultations were hardly private. There were 45 hunger strikers lying on mattresses close to each other, medical details and information were easily overheard by neighbors.

The health professionals told they were exposed to a lot of stories of suffering. The undocumented migrants faced multiple problems, after surviving in Belgium for years without access to legal jobs or help from the government. They shared stories of homelessness, chronic untreated medical problems, exploitation,... Providing social or medical care was very complicated in this context of limited means. This was further exacerbated by technical problems. Since the activists were squatting a building and not paying rent or water and electricity bills, there were frequent blackouts. Health professionals had to use candles or flashlights during their visits. Moreover, the activists prioritized their demands for residence permits over their own health, and sometimes refused medical care such as taking medicines or vitamins. In this context all health professionals wondered if simply being present and providing medical advice was enough. They also wondered how far they should go in their commitment to the struggle of the undocumented migrants. A medicine student argued:

*I limit my commitment to the medical side and I can indeed issue a medical report that the press can see, but it stops there. As a doctor, demonstrating with them and asking for papers for people whose background you do not know, that is a step too far (Medical student)*.

This quote illustrates the ambiguous position of medical care during a hunger strike. On the one hand they risk losing legitimacy in the eyes of the activists if they are not showing explicit support to their case, for example by joining demonstrations. A medical doctor remembered that she had been criticized because she did not participate in a rally organized by the undocumented migrants. On the other hand, they feared losing their credibility as a health professional in the press if they did more than providing medical care. This is also illustrated by another quote:

*If we don't do a good job, we will lose our credibility as health professionals: so we make weight charts. As a researcher I describe what I see and make a medical report, but that is as far as I go (Junior doctor)*.

Hunger strikes are a politically sensitive subject. The two quotes above illustrate that the health professionals are very aware of the presence of the press. They wondered on how they were being perceived and faced challenges on what to communicate and to whom. They were afraid of being discredited for not caring well or for not providing correct information on weight loss.

### Disagreements With the Patients

A recurrent theme during the discussions, was how to act where there was disagreement with decisions taken by the patient or when patients did not accept the offered treatment (cf. supra). A poignant example was one medical doctor who criticized a new mother who was breastfeeding a two-month-old baby for participating in the hunger strike. The doctor felt also responsible for the child and argued that the mother was unlikely to be able to take good care of her child. This illustrates the limitations healthcare workers experience of such individualized approaches to autonomy or responsibility. Where there were disagreements, the health professionals usually respected the autonomy of the patient, as is illustrated by the following quote:

*We don't approve of the strike, but we let them take responsibility for what they're doing to achieve their purpose (Medical doctor)*.

In line with this, many health professionals did feel concerned about the way group dynamics and group pressures impacted individual decision making. As an illustration of this, they mentioned a major dispute concerning the intake of vitamins, which were prescribed as part of the medical assistance.

*It was better not to mention that they were vitamins because vitamins are associated with food, but to say that we were preventing paranesthesia and ultimately relieving pain. Then they started to take them and there was a reverse group effect, I mean, the ones who took the vitamins explained to the others why they were doings so and everyone agreed. They felt relieved that everybody could take them (Junior doctor)*.

Initially, the spokesperson of the group had argued against vitamin intake because he was convinced that they had an important caloric input and that the activists therefore risked being accused of not really being on a hunger strike. The doctor explains how he managed to convince the group to take the vitamins, by explaining that they didn't contain calories and were important for the regulation of bodily functions. Because of lack of formal education the health literacy level of the participants was low and health professionals had to find creative ways to explain how to prevent irreversible, irreparable bodily harm. Once the doctor had managed to convince a few undocumented migrants on hunger strike, the others followed. Yet, the group pressure could also evolve in the other direction. Several health professionals were afraid that if one of the activists decided to stop drinking, everybody would follow because they did not want to lose face and tried to be as radical as each other. Some health professionals attributed this group pressure to cultural traditions, for example by referring to the strong family ties in non-western cultures. Others attributed it specifically to the hunger strike, describing as an essential feature for a hunger strike to be successful:

*The group feeling has to be very strong because, I think, you never do a hunger strike all by yourself (Junior doctor)*.

The disagreements with the patients made some of the health professionals wonder if the undocumented migrants on hunger strike in fact were interested in their advice or their findings:

*Had the request for medical assistance really come from them? Or from outside? Because I sometimes had the impression that they weren't interested in their own medical developments (Nurse)*.

This disinterest made the nurse wonder whether the undocumented migrants really had asked for the medical advice, or whether it was imposed from the outside and it is not entirely clear from the quote whether she wonders if, by providing medical assistance, she has become a tool for surveillance.

### Coping Strategies

The above-mentioned concerns about their own professional role and the difficult relationship with the patients were a major cause of stress for the health professionals. This is powerfully illustrated by the following quote:

*The first time I arrived back home, I started crying and I did not know why, I was all alone at home, everyone was gone and then, yes,… I was wondering what had to happen before you started doing that and yes, it was very intense.. I do not know how to explain what I learned but it was something, but I do not know what (Junior doctor)*.

The junior doctor describes how emotional she was due to the intensity of the first consultation. This seems in part attributed to the lack of immediate support. She describes the experience as a learning moment, but she also describes the hunger strike as something she is unable to grasp fully. Other health professionals shared they had nightmares about the strike and wondered if they were the only ones who had been overwhelmed by the situation.

Many health professionals expressed that, after leaving a consultation, they had hoped that the strike soon would end, so that they would not have to go back. They wanted to stay away, but eventually they went back anyway.

*A strange feeling is that every time I left, I wanted them to stop … I really wanted to receive an e-mail the next day that said “ah, by the way, they stopped” (Junior doctor)*.

One medical doctor felt relief when returning to her daily job where helping others was much easier. Others describe how they changed the way they cared:

*I was not really myself, but I was not really a caregiver either, I was a person who was putting up barriers to avoid being overly affected by what was going on, someone who does what has to be done but where that is detached from myself and my role as a caregiver (Medical student)*.

This medical student describes herself as a different person during the consultation at the hunger strike. She is not a caregiver, nor herself. She became a person who had to protect herself and avoid being affected too much. This is described as a psychological process of putting barriers and emotional detachment. Others described how they performed their consultations like robots: when they were asked to do something, they did it “mechanically”.

Many felt paralyzed by witnessing so much misery. They thought their medical knowledge had fallen short: they saw no way to alleviate the suffering. Some of them ended their commitment and stopped coming to the occupation. Others wrote testimonies on social media or talked to friends about their experience and invited them to also participate in the medical follow-up.

### Improving the Future Medical Monitoring of Hunger Strikes

The health professionals made a range suggestions for improving medical monitoring during hunger strikes.

According to the respondents, the team of health professionals should be multidisciplinary, they consider the presence of a psychologist to be essential, but they also mention the expertise of dieticians, internal medicine physicians, psychiatrists, and nurses. The team should meet prior to starting the consultations at the hunger strike, and this for at least two reasons. Firstly, it allows medical training on international guidelines for medical monitoring during a hunger strike such as around the short- and long-term consequences of fasting, medication use, vitamin intake, weight monitoring… Second, it means that younger health professionals can be mentored by colleagues who have previous experience with medical monitoring during a hunger strike around the emotional and professionals challenges.

Furthermore, the respondents stated that at the start of the consultations, it is important to make agreements with the spokesperson and the participants to the hunger strike in order to have a clear mandate for providing care and to clarify the role of the medical advice (to take vitamins or medication), i.e., treating symptoms and preventing the short- and long-term consequences of fasting. In order to avoid that patients are pressurized into continuing the hunger strike and refusing treatments due to group dynamics, our respondents also advised that consultations should be held in a separate room, where privacy is guaranteed. Some respondents argued that those on hunger strike should write down, in their own words, how they want to be treated if they go into a coma. But others were more hesitant:

*Wouldn't it be useful, if there is another strike, for a psychologist to come and talk to them about how far they want to go. Until death… But they should discuss this with a doctor, and regularly, because I think the idea that ‘we want to continue until we die' changes in the course of the strike (Medical doctor)*.

This quote shows that the doctor has doubts as to who is best suited to make this assessment: the doctor or the psychologist. This concern stems, among other things, from the possible neurological sequalae and confusion that might appear toward the last stage of a hunger strike. But more importantly, the quote describes the assessment of the “will to die” as an ongoing process, because it can change in the course of the strike.

Furthermore, the respondents stressed the importance of weekly medical meetings and regular email updates amongst the team during the hunger strike, but also the importance of good communication with the referral hospital, and a clear strategy on how to communicate with the press. At the end of the strike, an effort should be made to find a family physician for every participant to the hunger strike. A medical report with information about the necessary aftercare of a hunger strike should be made so that the family physician will be able to provide the medical follow-up. Lastly, the respondents agreed that once the hunger strike has ended, having a debriefing for the health professionals is highly recommended, both in order to evaluate the care that was delivered and in order to share the emotional challenges that were experienced.

## Discussion

This study provides insights into the experiences of health professionals while providing medical assistance during a hunger strike by undocumented migrants in a non-custodial setting. Our findings point to the complex relationship between the patient and the healthcare provider caused, in part, by group pressures and a lack of privacy. The findings also illustrate the (fear of) possible instrumentalization of the health professionals and the possible politicization of healthcare delivery during a hunger strike. Lastly, this study shows the psychological problems that can appear among health professionals providing health care during a hunger strike.

The health professionals providing medical assistance during the hunger strike did so because of a mix of fascination, curiosity and political sympathy. However, for most health professionals the reality proved to be challenging. Even though our respondents respected the decision of the activists to stop eating, many had conflicting feelings around it. Firstly, they questioned whether this decision was in fact a truly autonomous decision and these doubts were exacerbated by several experiences during the hunger strike. As our findings illustrate, “peer pressure” and internal group dynamics impacted on the willingness of individuals to follow medical advice, and even if the decision not to eat was an autonomous decision, they doubted whether it was a rational decision but the respondents struggled to find words to express these doubts about the patient's decision. Existing concepts in the literature, whether it be “noncompliant”, “nonadherent” or “suicidal”, all fall short of describing the decision to start a hunger strike. A framework to analyze these problems can be found in the Groupthink Theory, developed in 1972 by Irving L. Janis, The Groupthink Theory describes “*a deterioration of mental efficiency, reality testing, and moral judgment that results from in-group pressures*” (30). This happens especially in groups “*when they are highly cohesive, insulated from experts, perform limited search and appraisal of information, operate under directed leadership and experience conditions of high stress with low self-esteem and little hope of finding a better solution to a pressing problem than that favored by the leader or influential members*” (31). This theory also explains why it is difficult for a member to have a dissident opinion that can lead to exclusion from the group and in the case of a hunger strike, no access to the highly desired residence permit.

Secondly, during the hunger strike the health professionals questioned their own role as health professionals. Their approach primarily was an approach that can be described as “engaged presence” (32). However, this approach conflicted with more interventionist attitudes, and lead to difficult ethical dilemmas. Our findings illustrate that the complexity of the context made it impossible to practice “state-of-the-art” medicine In other contexts, being a health professional means having medical knowledge and acting upon it. Yet, in this situation the knowledge that the patient would have to eat to improve, cannot be acted upon. The health professional's deontological duty is to provide care, to do good and to do no harm. Instead, they were forced to simply respect their autonomy and watch them deteriorate. Moreover, the health professionals lacked thorough medical knowledge on how to treat symptoms caused by long term fastening, which enhanced insecurities about their own work. The respondents also expressed being uncomfortable around the lack of privacy during the medical visits. Privacy is an important issue while taking care of patients: it ensures that patients are able to make their personal decisions, that they are approached and treated as individuals, with respect and dignity. It ensures a more fruitful communication and is therefore essential for the quality of care and for guaranteeing professional secrecy. It was not easy for the health professionals to lower their expectations and accept the lack of privacy. Lastly, toward the end of the hunger strike the health professionals felt conflicted on whether the activists were still competent to make their own decisions, as the last phase of a hunger strike is characterized by neurological symptoms and confusion.

These two elements made it highly difficult to establish a “normal” functional therapeutic relationship between the undocumented migrants and the health professionals. The health professionals were confronted with patients asking for help, but subsequently often did not want to follow the medical advice. The therapeutic relationship was also complicated by the interference of several “third parties”, such as the spokesperson of the hunger strike, the press and government officials, who all had interest in and wanted information about the health status of the undocumented migrants on hunger strike. Our findings show that medical information, such as medical reports about weight loss or deterioration, even when formulated in the most neutral possible way, had a political value for these third parties and played a role in negotiations between the activists and the migration authorities. Medical information (e.g., about severe, life-threatening weight-loss) can be used to put pressure on the government (5). In this context of politicization, the health professionals were particularly concerned that the therapeutic relationship could take on a merely instrumental character, but also that they risked losing their professional credibility.

This potential instrumentalization of the therapeutic relationship has also been described in literature on humanitarian aid to undocumented migrants (33, 34) and in hunger strikes in custodial settings (35). In custodial settings researchers have mainly pointed to the dual loyalty in which the health professionals are caught (36–40). They have to find a balance between obeying the orders of their employer and the authorities on the one hand and taking the side of those on hunger strike on the other. Our findings show that, compared to custodial settings, the potential instrumentalization is more multi-dimensional in a non-custodial setting. The health professionals expressed that they did not only have to cope with potential instrumentalization by the government, they also felt instrumentalized by those on hunger strike. While preferring to stick to a humanitarian, medical role, the medical aid they offered was framed as a support for the political demands of the undocumented migrants. They felt pushed to speak to the press or participate in rallies, even though they did not wish to become spokespersons on behalf of the activists. On the other hand, the health professionals also perceived distrust from the undocumented migrants. Our findings show that several of the undocumented migrants on hunger strike were not interested in medical monitoring. This might be explained by the perception that the health professionals were there to control and verify whether the activists really were not eating. Having lived as undocumented in Belgium for many years, having very poor access to health care and sometimes having been rejected or mistreated while seeking medical care, might also have contributed to the suspicion regarding the intentions of the volunteering health professionals.

In order to overcome these barriers and build a relationship of mutual trust, the health professionals tried to claim a neutral, medical and humanitarian role, even though their initial engagement was, amongst other things, motivated by political sympathies. They mainly focused on preventing irreversible health problems. They showed interest in the reasons behind the refusal to eat or to comply with medical advice, they took the group dynamics into account, but they also sometimes engaged in frank discussions when disagreeing with patient's decisions. However, as our findings show, for most health professionals their position remained one that was full of ambiguities throughout the follow-up of the hunger strike. The few health professionals who publicly defended the demands of the hunger strikers did this after thorough discussion with the undocumented migrants, often because they also had already engaged in other political or union actions before.

Although health professionals' exposure to the suffering patients and activist was short, the focus groups discussions revealed symptoms of psychological distress and behavioral adaptations amongst the health professionals. These included nightmares, re-experiencing the home visits, avoiding returning to the hunger strike and self-doubt. Similar issues have been described in professionals and caregivers working with traumatized refugees coming from Latin America and North Korea (41, 42). Figley labeled this phenomenon as Secondary Traumatic Stress (STS): “*the natural consequent behaviors and emotions resulting from knowing about a traumatizing event experienced by a significant other. It is the stress resulting from helping or wanting to help a traumatized or suffering person”* (p. 7) (43). STS can present at psychological, behavioral, physical or professional level and include symptoms of intrusion or re-experiencing, avoidance and arousal symptoms (44–46). We noticed that medical students and less experienced junior doctors who participated in the medical assistance were especially vulnerable to these symptoms. This analysis was also made in a Romanian study where students who started studying medicine for altruistic reasons were more vulnerable to STS than fellow students who had chosen their field because they were more tempted by the respect and recognition the job would bring (47).

Our respondents made several suggestions for preventing STS and to improve medical assistance during a hunger strike. They stressed the importance of mentoring by health professionals with previous experience with medical assistance during a hunger strike and the importance of multidisciplinary teams, in particular the presence of a psychologist. They also advocated regular meetings, mutual communication and debriefing. In addition to these suggestions, we would like to make a case for more attention being paid to STS in the medical curriculum which as, to our knowledge, prevention of STS is currently not part of the curriculum. Several scholars have argued that health professionals should be made aware of the possibility, risk factors and first signs of STS (48, 49).

This study is the first one to report on how health professionals experience medical monitoring during a hunger strike in a non-custodial setting. This study disclosed the vulnerability and the highly complex position of health professionals in this setting. A potential weaknesses is the relatively small sample on which the findings are based and the lack of diagnostic tests to objectify the psychological distress of the respondents.

## Conclusion

Our study of a hunger strike among undocumented migrants in Belgium shows the complexities health professionals face when providing medical assistance of those on hunger strike. The health professionals were confronted with people who jeopardize their health for a higher goal. They were struck by the paradox of being willing to die in order to get a better life, of fighting while getting weaker. Their own position also became highly ambiguous. They were stuck between respecting the individual autonomy of and trying to prevent harm to the physical integrity of their patient. Moreover, the context of a hunger strike made the therapeutic relationship prone to external pressures. In this context, many health professionals feared that their presence or their work could be instrumentalized by the hunger strikers or the authorities. In order to overcome these ambiguities and create a therapeutic relationship of mutual trust the health professionals tried to claim a neutral, medical and humanitarian role.

After the hunger strike, several health professional reported symptoms indicative of STS. Therefore, our study makes several recommendations for improving medical assistance of hunger strikes and to protect the health professionals who engage in providing healthcare in such difficult circumstances.

## Data Availability Statement

The raw data supporting the conclusions of this article will be made available by the authors, without undue reservation.

## Ethics Statement

The research was approved by the Medical Ethics Committee UZ Brussel[s]—VUB (B.U.N. 143,201,940,934). All participants filled in and signed a consent form, and no participants received financial incentives. The participants provided their written informed consent to participate in this study.

## Author Contributions

RV, DL, FL, DD, and JV supervised the research and revised the different drafts of the article. All authors contributed to the manuscript revision, read, and approved the submitted version.

## Conflict of Interest

The authors declare that the research was conducted in the absence of any commercial or financial relationships that could be construed as a potential conflict of interest.

## Publisher's Note

All claims expressed in this article are solely those of the authors and do not necessarily represent those of their affiliated organizations, or those of the publisher, the editors and the reviewers. Any product that may be evaluated in this article, or claim that may be made by its manufacturer, is not guaranteed or endorsed by the publisher.
